# Editorial: B Cell Non-Hodgkin’s Lymphoma & Tumor Microenvironment Crosstalk: An Epigenetic Matter?

**DOI:** 10.3389/fgene.2022.912737

**Published:** 2022-05-19

**Authors:** Y Denizot, MS Braza, R Amin

**Affiliations:** ^1^ UMR CNRS 7276, INSERM U1262, Equipe Labellise LIGUE 2018, Universite de Limoges, CBRS, Limoges, France; ^2^ Department of Oncological Sciences, Icahn School of Medicine at Mount Sinai, New York, NY, United States; ^3^ Department of Biochemistry, University of Nebraska at Lincoln, Lincoln, NE, United States

**Keywords:** B lymphoma, tumor microenvironment, NHL—Non-Hodgkin lymphomas, immune cells, Epigenetic alteration

Non-Hodgkin B Lymphoma (B-NHL) is the seventh most common cancer of the lymphatic system. Over 40 subtypes of B-NHL have been phenotypically characterized, and the two most common subtypes are diffuse large B-cell lymphoma (DLBCL) and follicular lymphoma (FL). These two subtypes alone account for approximately 60% of all cases. One of the greatest challenges in B-NHL is that a notable percentage of patients (20–30% of cases) relapse and have a refractory response to the latest immunochemotherapy treatments (Siegel et al., 2020; Marofi et al., 2021). The complexity of treating cancer like B-NHL is the result of high inter-and intratumoral heterogeneity which supports subclonal evolution, disease progression, and resistance (Schürch et al., 2018).

B-NHL results from a serial accumulation of genetic aberrations that largely target chromatin-modifiers to orchestrate lymphomagenesis (Esmeray and Küçük, 2020; Amin and Braza, 2022; Ma et al., 2022). Each regulator that is assigned with specific genetic mutations will result in a particular epigenetic landscape, thereby acting as a “writer” or “eraser” of gene expression. Moreover, growing evidence suggests that intratumoral heterogeneity mutually influences the genetic landscape and the tumor microenvironment (TME), especially with respect to tumor initiation, immune escape, tumor transformation, and degree of refractoriness (Scott and Gascoyne, 2014; Tarte, 2017; Steen et al., 2021). The tumor ecosystem is a dynamic niche composed of tumor cells, non-malignant immune cells, and stromal cells that communicate through a variety of complex biological processes (Scott and Gascoyne, 2014; Ochando and Braza, 2017; Tarte, 2017; Mourcin et al., 2021). Although the complex connectivity between the genetic landscape and tumor ecosystems is not completely understood, tailoring treatment to the epigenetic or TME component is currently receiving the greatest interest as a potential druggable target to abrogate microenvironmental support and improve anti-tumor responses (Ribeiro et al., 2019; Liu et al., 2021; Marofi et al., 2021).

The purpose of our research topic is to illustrate the epigenetic language that influences the tumor microenvironment in B-NHL tumorigenesis. We received two original research manuscripts and two outstanding reviews describing the current literature and clinical advancements in B-NHL treatments.

Considering the essential role of inflammation in most cancers, Wang et al. explored the landscape of pyroptosis-related genes (PRGs), which play a role in inflammatory programmed cell death in DLBCL. The authors performed a comprehensive analysis of 52 PRGs based on publicly available transcriptome datasets from DLBCL patients. This integrative study showed that the inflammatory signature inversely correlates with the highest complete remission rate. Specifically, low inflammation was characterized by low immune- and stromal cell infiltration, while high inflammation was characterized by enriched stromal infiltration, which also corresponded with a poor prognosis. By combining the pyroptosis gene signature and clinical characteristics, the authors built a predictive nomogram to evaluate overall survival in DLBCL patients and found that the pyroptosis risk score could significantly differentiate between high and low-risk DLBCL patients. Taken together, the pyroptosis risk score and the PRGs offer interesting tools that can be used as prognostic markers and serve as potential therapeutic targets to improve DLBCL treatment outcomes.

In addition, miRNAs, a class of non-coding small RNA, contribute to B-NHL pathogenesis by regulating mRNA translation and function (Getaneh et al., 2019; Fuertes et al., 2020). Elliot et al. investigated the level of DNA methylation of miRNA-17-92 clusters and their target the tumor suppressor gene, *TET2*, in different NHL subtypes. The authors found an increase of aberrant CpG methylation site in the regulatory region of *TET2* and miR17-92a cluster in B-NHL compared to healthy controls; however, the study didn’t reveal a significant association between them. The clear discrepancy in DNA methylation of promoter regions between cohorts offers additional mechanisms to explore for B-NHL pathogenesis and therapeutic design.

The two review articles provide an extensive update of chromatin modifiers as key drivers in shaping immune evasion and TME niches. Focusing on DLBCL and FL, Mondello et al. reviewed each chromatin-modifying enzyme and their competitive interplay in connecting molecular events in the immune system with features of the TME. The authors also discussed potential therapeutic strategies in tailoring cancer treatments to epigenetic modifiers to improve immunotherapy approaches and reduce the epigenetic influence on TME niches.

Serganova et al. expanded the literature on DLBCL and FL by reviewing the tripartite connection between metabolic, epigenetic, and immune ecosystems. The authors beautifully illustrated various metabolic circuits’ that influence chromatin-modifying enzyme activity. Mapping different metabolic features demonstrated the plasticity of metabolic pathways that are borrowed by B-NHL to fuel survival, resistance, and immunosuppression. The authors also summarized the latest clinical trials and drug therapies with a focus on epigenetic inhibitors, potential anti-metabolic agents, and immunotherapy. The comprehensive investigation discussed in this review addresses the complex interaction between scales in B-NHL and the future of therapeutic design in developing persuasive anti-tumoral responses.

Although the landscape of epigenetic alteration and its ecosystems is broadly understood, critical questions remain regarding the impact of mutational heterogeneity and the integrative connection between distinct biological processes contributing to B-NHL lymphomagenesis. Multidisciplinary approaches that combine experimental investigation with systems biology approaches will likely improve our understanding of the complex interaction between biological phenomena at the system level and reverse therapy resistance. The contribution of our research topic provides insight into the heterogeneity of malignancies and their ecosystem paving the way for the development of more personalized next-generation therapies.
